# Prophylactic full-thickness suturing of the rectus abdominis muscle reduces parastomal hernia after enterostomy: a 5-year retrospective cohort study

**DOI:** 10.3389/fmed.2026.1978158

**Published:** 2026-10-07

**Authors:** Guoqiang Zhang, Bowen Zheng

**Affiliations:** 1 Department of Colorectal and Anal Surgery, Shengzhou People's Hospital (Shengzhou Branch of the First Affiliated Hospital of Zhejiang University School of Medicine, Shengzhou Hospital of Shaoxing University), Shaoxing, China; 2 Ningbo University, Ningbo, China

**Keywords:** full-thickness suture, intestinal stoma, parastomal hernia, prevention, rectus abdominis reinforcement

## Abstract

**Background:**

Parastomal hernia (PSH) is one of the most common long-term complications after enterostomy. Prophylactic mesh can reduce PSH risk but is limited by mesh-related adverse events and high costs. This study aimed to evaluate the efficacy and safety of prophylactic full-thickness rectus abdominis suturing for PSH prevention without implant materials.

**Methods:**

This single-center retrospective cohort study enrolled 244 patients undergoing enterostomy from January 2020 to December 2024. Patients were allocated alternately by calendar-month: 94 patients received full-thickness rectus-suture reinforcement (reinforcement group), and 150 underwent conventional stoma creation (conventional group). The primary endpoint was CT-confirmed PSH incidence within 2-year follow-up. Secondary endpoints included hernia-sac diameter, time-to-PSH-onset and stoma-related complications. Univariable and multivariable logistic regression, Firth penalized regression, 1:1 propensity-score-matching (PSM), Kaplan–Meier survival analysis and interrupted time-series (ITS) segmented regression were performed.

**Results:**

The reinforcement group showed significantly lower 2-year PSH incidence (9.6% vs. 27.3%, *P* < 0.001). Suture reinforcement was an independent protective factor against PSH (adjusted OR = 0.29, 95% CI: 0.13–0.65, *P* = 0.002), and Firth-penalized logistic regression confirmed the stability of this finding (OR = 0.31, 95% CI: 0.14–0.68, *P* = 0.003). After PSM, the inter-group difference remained significant (14.9% vs. 31.9%, *P* = 0.006), and multivariable regression in the matched cohort still identified suture reinforcement as an independent protective factor (adjusted OR = 0.35, 95% CI: 0.17–0.72, *P* = 0.004). Patients in the reinforcement group had smaller hernia-sac diameter (2.9 cm vs. 7.3 cm, *P* < 0.001) and delayed PSH onset (12.2 months vs. 6.7 months, *P* = 0.001). ITS analysis supported the robustness of observational findings. Early postoperative complication rates were comparable between groups.

**Conclusions:**

Prophylactic full-thickness rectus-abdominis suturing is associated with reduced PSH incidence, delayed hernia onset and milder hernia severity. This implant-free, low-cost technique is safe and simple. Prospective randomized trials are needed to further validate its clinical value.

## 1 Introduction

Intestinal ostomy is a common procedure in colorectal surgery, and parastomal hernia (PSH) is one of its most frequent long-term complications. The reported incidence of PSH during postoperative follow-up ranges from 30% to 50% (1–3). Among symptomatic patients, approximately 20%–30% require surgical intervention, and postoperative recurrence rates remain high (4, 5). PSH not only impairs stoma care and patients' quality of life but may also lead to serious complications such as bowel incarceration and obstruction, substantially increasing healthcare resource utilization (6).

To reduce PSH incidence, various preventive techniques have been proposed, of which prophylactic mesh reinforcement is the most extensively studied. Although meta-analyses have shown that mesh reinforcement lowers PSH risk (7), associated complications—including infection, intestinal erosion, and chronic pain—and high costs limit its widespread clinical adoption (8, 9). Other preventive strategies, such as optimized stoma site selection or postoperative abdominal binders, offer limited efficacy or lack support from high-quality evidence (10). Thus, the development of simple, cost-effective, and safe preventive measures is of considerable clinical importance.

The rectus abdominis complex constitutes the core anatomical structure maintaining abdominal wall tension (11). During intestinal ostomy creation, the bowel traverses this complex, disrupting its integrity and creating weak zones at the superior and inferior poles of the stoma that are vulnerable to elevated intra-abdominal pressure. As intra-abdominal pressure increases, the stomal defect may gradually expand along the rectus muscle fibers, leading to PSH formation (12). A fundamental principle in PSH repair is to close this defect using non-absorbable sutures to reduce the fascial gap (13). Accordingly, reinforcing the superior and inferior poles of the stomal aperture with full-thickness sutures at the time of stoma formation should theoretically enhance resistance to intra-abdominal pressure, thereby preventing progressive stomal dilatation and subsequent PSH. Although the concept of suture reinforcement is not novel, the standardized use of full-thickness, non-absorbable sutures specifically at the stomal poles has rarely been systematically reported in the literature. For this reason, we conducted a 5-year retrospective cohort study to assess the clinical safety and efficacy of this technique, analyze its impact on PSH incidence and timing, and explore its feasibility, with the goal of establishing a simple, cost-effective, and effective strategy for PSH prevention.

## 2 Materials and methods

### 2.1 General data

A retrospective analysis was conducted on clinical data of patients who underwent enterostomy in our department from January 2020 to December 2024. Group allocation was implemented by a pre-specified non-randomized alternating-month schedule throughout the study period. This procedure was not formal randomization. Patients undergoing enterostomy in odd-numbered calendar months (January, March, May, July, September, November) received conventional stoma creation (conventional group). Patients operated on in even-numbered calendar months (February, April, June, August, October, December) underwent prophylactic full-thickness rectus-abdominis suture reinforcement (reinforcement group). All participating surgeons were proficient in both operative techniques, and the assigned surgical procedure was strictly implemented regardless of individual-patient-risk-profile. Intra-group cross-over was not permitted. The unequal sample-size was entirely caused by fluctuating monthly case-volume rather than investigator-driven selection.

### 2.2 Inclusion criteria

Inclusion criteria were: (1) age ≥ 18 years; (2) ileostomy or colostomy; and (3) enterostomy performed at our hospital between January 2020 and December 2024.

### 2.3 Exclusion criteria

Exclusion criteria were: (1) emergency surgery for multiple trauma that could affect wound healing; (2) history of midline incisional hernia or parastomal hernia; (3) postoperative bowel obstruction caused by tumor recurrence that could interfere with follow-up assessment; and (4) loss to follow-up or incomplete clinical data.

### 2.4 Surgical methods

#### 2.4.1 Reinforcement group

A circular or elliptical skin incision (approximately 2 cm in diameter) was made at the predetermined stoma site. After skin excision, the anterior rectus sheath was incised, the rectus abdominis muscle fibers were split bluntly, and the posterior rectus sheath together with the peritoneum was opened to enter the peritoneal cavity. The stomal fascial aperture was adjusted to 1.5–2.0 cm. In emergency cases with bowel edema, the aperture was tailored to the smallest size that allowed tension-free exteriorization. For full-thickness reinforcement, either 1–0 Prolene (monofilament polypropylene, non-absorbable) or No. 7 silk suture (braided, non-absorbable; approximate diameter 0.5–0.6 mm; institutional designation) was used. Full-thickness sutures incorporating the anterior rectus sheath, rectus abdominis, posterior rectus sheath, and peritoneum were pre-placed at the superior and inferior poles of the stoma aperture and left untied. For a loop stoma, the intestinal loop was exteriorized through the aperture; for an end stoma, the bowel end was exteriorized. After confirming adequate bowel viability and absence of tension on the exteriorized bowel, the inferior suture was tied first, followed by the superior suture. The final stomal lumen admitted one small finger (approximately 1.5 cm). Standard stoma maturation was subsequently performed according to stoma type: for a loop stoma, the proximal and distal limbs were matured; for an end stoma, the single bowel end was matured (Figures 1, 2).

**Figure 1:**
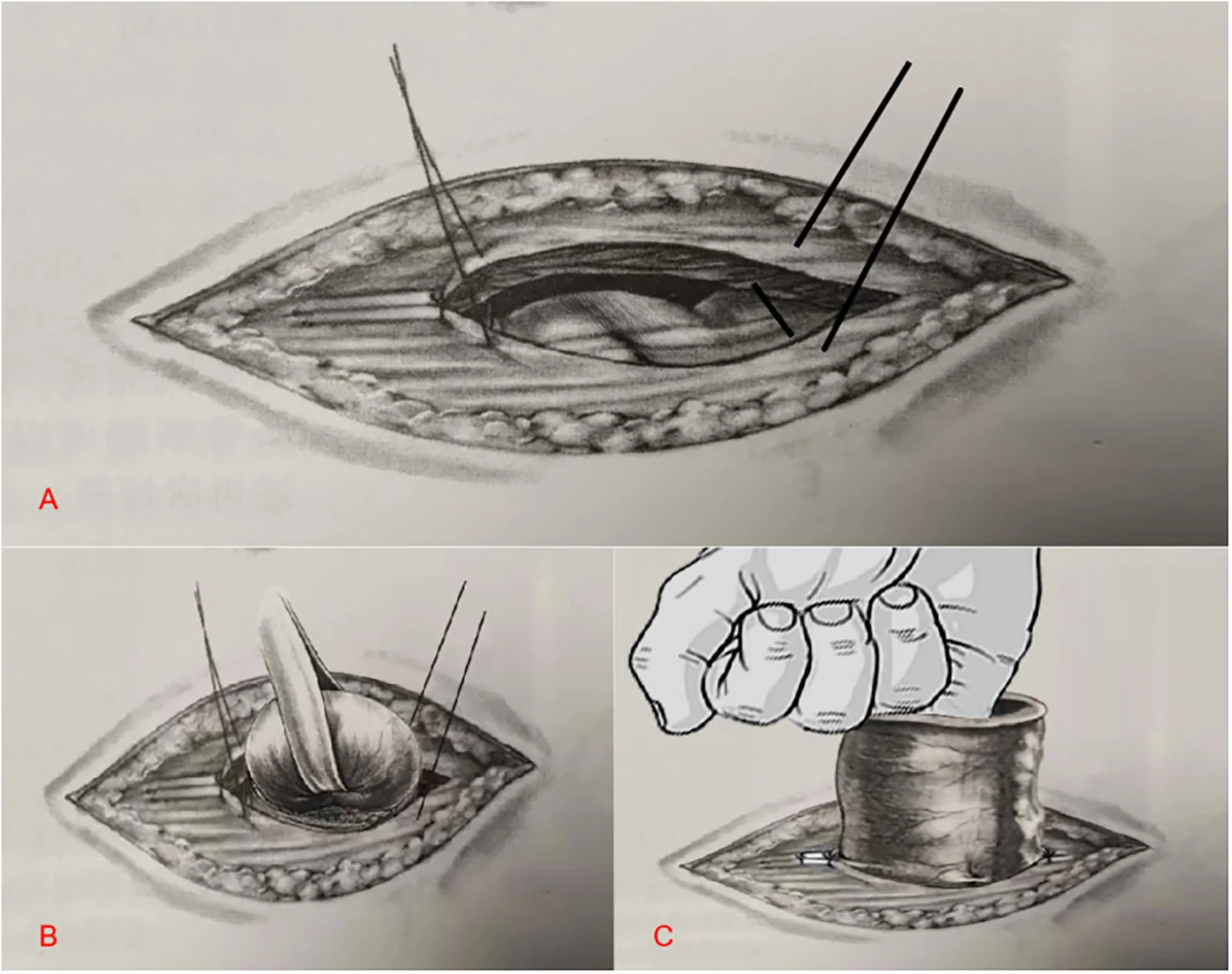
Schematic diagram of the suture reinforcement group procedure **A:** After incising the peritoneum and establishing the intestinal stoma, one to two preplaced sutures are passed through the full thickness of the rectus abdominis muscle at both the upper and lower ends of the stoma site; the sutures are temporarily left untied. **B:** Loop stoma, exteriorizing the bowel. **C:** End stoma, after exteriorizing the bowel, the sutures are tied, ensuring that a small finger can pass through the intestinal lumen.

**Figure 2:**
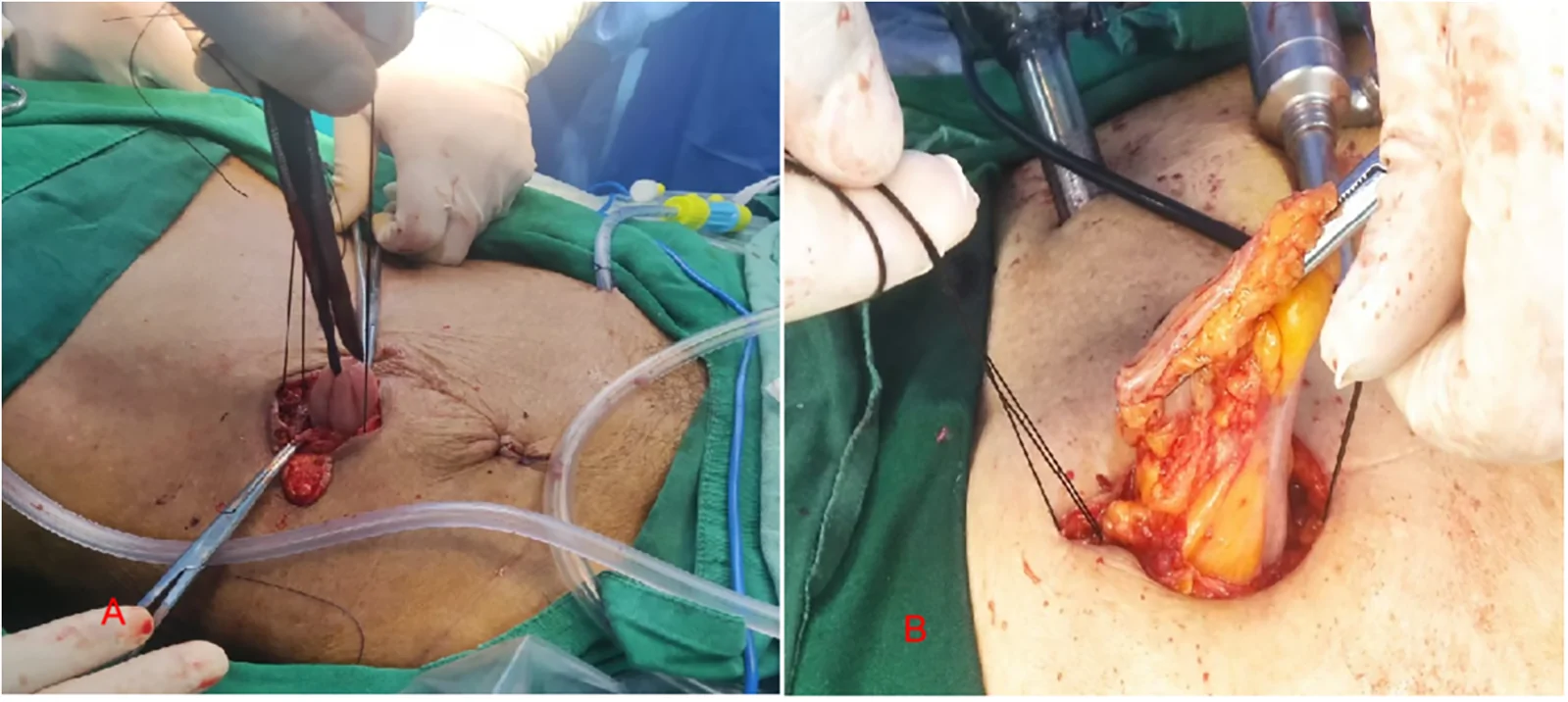
Schematic diagram of the procedure in the suture reinforcement group. **(A)** (Loop stoma) and **(B)** (End stoma): ① Preplacement and fixation stage: The intestinal segment **(A)**: the intestinal loop temporarily secured with a marking tape; **(B)**: the end stoma bowel segment after exteriorization and length confirmation) is positioned intra-abdominally, and pre-reinforcement sutures are placed through the full thickness of the rectus abdominis muscle at both the upper and lower ends of the stoma site. ② Final formation stage: After confirming that the stoma size is appropriate, the preplaced sutures are tied to complete the stoma construction.

#### 2.4.2 Conventional group

Stoma location and layered dissection were identical to those in the reinforcement group. After entry into the peritoneal cavity, the fascial aperture was adjusted to 1.5–2.0 cm using the same criteria as in the reinforcement group. For a loop stoma, the intestinal loop was exteriorized; for an end stoma, the bowel end was exteriorized. Stoma maturation sutures were then placed routinely. The final stomal lumen also admitted one small finger.

### 2.5 Data collection

Baseline patient data were collected, including age, sex, nutritional status, body mass index (BMI), smoking, neoadjuvant ± adjuvant therapy(neoadjuvant therapy was defined as preoperative chemotherapy and/or radiotherapy, and adjuvant therapy as postoperative chemotherapy and/or radiotherapy) and comorbidities (diabetes mellitus, chronic respiratory disease, and malignancy). Operative parameters comprised surgical approach (open vs. laparoscopic), timing of surgery (elective vs. emergency), suture material (silk No. 7 vs. 1-0 Prolene), stoma configuration (end vs. loop), and stoma site.

### 2.6 Follow-up and imaging evaluation

All patients within our institutional stoma cohort received scheduled contrast-enhanced abdominal CT examinations according to standardized follow-up protocols at 6, 12, and 24 months postoperatively. Additional contrast-enhanced CT was arranged promptly whenever clinical manifestations suggested possible PSH. Postoperative abdominal CT served as the primary modality for diagnosing PSH and measuring hernia sac diameter. The data cutoff date was June 2026. Follow-up information was supplemented by outpatient interview and telephone contact until stoma closure or completion of the 2-year postoperative observation period to record PSH events. Patients enrolled between July and December 2024 had not completed 24 months of follow-up by the data cutoff date. For these patients, available follow-up data up to the data cutoff date were analyzed, and they were censored at the last available CT assessment in the Kaplan–Meier time-to-event analysis. For the chi-square test–based 2-year primary incidence analysis, these patients were not included in the calculation of 2-year PSH incidence to avoid misclassification due to insufficient follow-up.

### 2.7 Definitions of loss to follow-up and incomplete data

Loss-to-follow-up was defined as failure to complete scheduled imaging-assessment within predefined time-windows (6-, 12-, 24-month), and patients unreachable by telephone or outpatient-clinic consultation. Incomplete-data indicated absence of critical covariates (BMI, comorbidity information, operative-records), which precluded observational-statistical-analysis.

### 2.8 Outcome definition and censoring

The primary outcome indicator was CT-verified PSH occurrence within 2 years after surgery. For primary incidence analysis using the chi-square test: patients who underwent stoma reversal before the 2-year endpoint without developing PSH were not counted as PSH events and were treated as non-PSH cases according to the as-treated observational analytical strategy. Patients enrolled between July and December 2024 who had not reached the 24-month endpoint were not included in the chi-square test–based 2-year PSH incidence calculation. For Kaplan–Meier time-to-event analysis: patients who could not complete 2-year observation due to tumor recurrence, stoma reversal, or prior PSH repair surgery were regarded as censored cases. Patients with incomplete 24-month follow-up were censored at their last available CT assessment.

### 2.9 Diagnostic criteria for parastomal hernia

PSH diagnosis complied with 2014 European Hernia-Society classification and relevant guidelines. Imaging-diagnostic-criterion: abdominal plain or contrast-enhanced-CT (slice-thickness ≤ 5 mm) demonstrating abdominal-viscera (bowel, omentum etc.) protruding outside fascial plane adjacent to the stoma. All CT-images were independently assessed by two radiologists; inter-rater-agreement was evaluated by Kappa-statistic, with pre-specified Kappa > 0.80 representing satisfactory diagnostic-consistency.

Measurement protocol for hernia-sac dimension: on axial and coronal CT-reconstruction images, transverse-diameter and anteroposterior-diameter of fascial-defect were measured; maximum-diameter of hernia-sac was recorded. All measurements adopted built-in electronic-measurement-tools within Picture Archiving and Communication System (PACS).

Two attending-level radiologists (≥ 5-years experience in abdominal-imaging) accomplished initial image-screening, index-measurement and data-recording. When disagreement or ambiguous-findings occurred, one associate-chief-surgeon specialized in abdominal-wall-surgery participated in joint-review. If consensus could still not be achieved between two radiologists, senior radiology or general-surgery consultants would perform joint-assessment.

Physical-examination served only as auxiliary-diagnostic-method. In accordance with European-Hernia-Society guidelines, all patients received standardized physical-assessment by stoma-therapists or surgeons during outpatient-follow-up; physical-signs including peristomal palpable mass, cough-impulse-sign were documented. When physical-examination findings contradicted CT-results, CT-diagnosis was prioritized.

### 2.10 Statistical analysis

Statistical analyses were performed using SPSS 31.0 and R 4.3.1. Continuous and categorical variables were compared using independent-samples t-test and *χ*^2^ test, respectively. Univariate logistic regression screened potential risk factors; variables with *P* < 0.10 were entered into multivariable models. Firth penalized regression was applied to reduce separation bias, and backward stepwise selection retained variables with *P* < 0.05. Model fit was assessed by the Hosmer-Lemeshow test. Adjusted odds ratios and 95% confidence intervals were reported.

Propensity score matching (1:1 nearest-neighbour, calipe*r* = 0.2 × SD of logit-transformed PS) was performed; balance was assessed by standardized mean difference (SMD < 0.10 indicated adequate balance). PSH-free survival was estimated by the Kaplan–Meier method with log-rank test.

Interrupted time-series segmented regression on monthly PSH incidence was used to address time-related confounding from alternating-month allocation:

*Yt* =*β*0 +*β*1 *Tt* +*β*2 *Xt* +*β*3 (*Tt* ×*Xt* )+*εt*, where *β*2*β*2 estimates immediate level change and *β*3*β*3 estimates slope change.

Model outputs including R^2^ and Durbin-Watson statistic are presented in Table 1.

**Table 1:** Interrupted Time-Series (ITS, Segmented Regression) Detailed Output.

Parameter	Estimate	95%CI	*P*-value
*β*0(Intercept)	0.221	0.147-0.295	<0.001
β1(Pre-intervention time slope)	0.0042	0.0011-0.0073	0.009
β2(Immediate level change after intervention)	-0.074	-0.142～-0.006	0.034
β3(Change of post-intervention slope)	-0.0031	-0.0058～-0.0004	0.026
Model goodness-0f-fit	R2 = 0.71	-	-
Durbin-Watson statistic	1.94	-	-

1. Monthly aggregated PSH incidence rate was adopted as dependent variable **Y_t_**.

2. **Y_t_**=β0+β1Tt+β2Xt+β3(Tt × Xt)+*ε*t **T_t_**:continuous time (1-60 months); **X_t_**:intervention indicator (0 = odd-month conventional group;1 = even-month suture-reinforcement group).

3. Durbin-Watson statistic=1.94 indicates negligible first-order autocorrelation of model residuals.

Subgroup analyses did not apply Firth penalized regression; subgroups with zero events should therefore be regarded as exploratory.

All tests were two-sided, with *P* < 0.05 considered statistically significant.

## 3 Results

### 3.1 Baseline characteristics and operative-outcomes

Among 244 patients (94 reinforcement, 150 conventional), baseline demographic characteristics, comorbidities, and operative parameters were well balanced (all *P* > 0.05). No deep surgical site infection or chronic sinus formation occurred regardless of suture material or smoking, with no intergroup difference; early postoperative complications were comparable between groups (all *P* > 0.05) (Table 2).

**Table 2:** Comparison of baseline characteristics and operative details between the reinforcement and conventional groups before and after propensity score matching.

Variable	Before matching				After matching			
	Total Cohort (*N* = 244)	Reinforcement Group (*n* = 94)	Conventional Group (*n* = 150)	*P*-Value	Reinforcement Group (*n* = 94)	Conventional Group (*n* = 94)	SMD	*P*-Value
Demographic characteristics								
Male, *n* (%)	147（60.2）	61（64.9）	86（57.3）	0.240	58（61.7）	56（59.6）	0.044	0.763
Age (years), mea*n* ± SD	71.4 ± 9.2	70.6 ± 9.0	71.9 ± 9.4	0.290	70.8 ± 9.1	71.2 ± 9.3	0.043	0.762
Nutritional status								
Serum albumin (g/L), mea*n* ± SD	33.1 ± 4.6	32.6 ± 4.6	33.4 ± 4.5	0.222	32.8 ± 4.5	33.1 ± 4.4	0.067	0.643
BMI（kg/m^2^）	26.4 ± 4.4	27 ± 5	26 ± 4	0.086	28 ± 5	27 ± 4	0.221	0.132
Comorbidities								
Diabetes mellitus, *n* (%)	68（27.9）	23（25.0）	45（30.0）	0.071	23（24.5）	25（26.6）	0.049	0.736
Chronic respiratory disease, *n* (%)	28（11.5）	12（12.8）	16（10.7）	0.617	12（12.8）	11（11.7）	0.032	0.823
Smoking, *n* (%)	104 (42.6)	42 (44.7)	62 (41.3)	0.608	42 (44.7)	39 (41.5)	0.065	0.659
Malignant tumors, *n* (%)	170（69.7）	68（72.3）	102（68.0）	0.473	68（72.3）	66（70.2）	0.047	0.748
Neoadjuvant ± Adjuvant therapy *n* (%)	137 (56.1)	52 (55.3)	85 (56.7)	0.893	52 (55.3)	55 (58.5)	0.065	0.772
Operative Details								
Surgical Approach, *n* (%)								
Open	127 (52.0)	54 (57.4)	73 (48.7)	0.312	53（56.4）	52（55.3）	0.022	0.846
Laparoscopic	117 (48.0)	40 (42.6)	77 (51.3)		41（43.6）	42（44.7）	0.022	
Stoma location, *n* (%)								
Upper Abdomen	15 (6.1)	7 (7.4)	8 (5.3)	0.504	7（7.4）	6（6.4）	0.041	0.820
Lower Abdomen	229 (93.9)	87 (92.6)	142 (94.7)		87（92.6）	88（93.6）	0.041	
Operative setting, *n* (%)								
Elective	199 (81.6)	73 (77.7)	126 (84.0)	0.214	73（77.7）	75（79.8）	0.052	0.713
Emergency	45 (18.4)	21 (22.3)	24 (16.0)		21（22.3）	19（20.2）	0.052	
Stoma configuration, *n* (%)								
End stoma	66 (27.0)	30 (31.9)	36 (24.0)	0.176	30（31.9）	28（29.8）	0.046	0.742
Loop stoma	178 (73.0)	64 (68.1)	114 (76.0)		64（68.1）	66（70.2）	0.046	
Stoma Segment, *n* (%)								
Small Bowel	166 (68.0)	63 (67.0)	103 (68.7)	0.789	63（67.0）	64（68.1）	0.023	0.865
Colon	78 (32.0)	31 (33.0)	47 (31.3)		31（33.0）	30（31.9）	0.023	
Suture								
NO.7 Silk, *n* (%)	142（58.2）	55（58.5）	87（58.0）	0.937	55（58.5）	54（57.4）	0.023	0.848
1-0 Prolene, *n* (%)	102（41.8）	39（41.5）	63（42.0）		39（41.5）	40（42.6）	0.023	

**SMD,** Standardized mean difference; SMD < 0.10 indicates that covariates were well balanced between the two groups.

Neither smoking nor neoadjuvant/adjuvant therapy was independently associated with PSH. Both were balanced before and after matching/PSM. In univariable analysis, neither was significant (smoking: OR = 0.97, 95% CI 0.52–1.82, *P* = 0.92; neoadjuvant/adjuvant therapy: OR = 0.60, 95% CI 0.32–1.12, *P* = 0.105; both *P* > 0.05). After forced inclusion, suture reinforcement remained an independent protective factor (aOR = 0.29, 95% CI 0.13–0.66 and 0.13–0.65, respectively; *P* = 0.002 for both), whereas neither smoking (aOR = 1.00, 95% CI 0.52–1.93, *P* = 0.99) nor neoadjuvant/adjuvant therapy (aOR = 0.75, 95% CI 0.37–1.52, *P* = 0.43) was independently associated with PSH. Subgroup analyses showed consistent protective effects (Tables 2–4).

**Table 3:** Univariable and multivariable logistic regression analysis of factors associated with parastomal hernia after enterostomy.

Variable			Univariate Analysis OR (95% CI)	Univariate *P*-value	Multivariate Analysis aOR (95% CI)	Multivariate *P*-value
Primary Exposure	Suture Reinforcement (Yes vs. No)		0.28 (0.13–0.59)	0.001	0.29 (0.13–0.65)	0.002
Patient-Related Factors	Male (vs. Female)		1.20 (0.75–1.92)	0.450	1.18 (0.72–1.94)	0.510
	BMI Category^a^	Moderate (vs. Low)	0.47 (0.15–1.46)	0.188	0.48 (0.15–1.50)	0.204
		High (vs. Low)	2.13 (1.02–4.46)	0.044	2.24 (1.06–4.76)	0.035
	Age (per 10-year increase)		1.15 (0.92–1.44)	0.220	1.12 (0.88–1.43)	0.340
	Smoking(vs. NO)		0.97 (0.52–1.82)	0.92	1.00 (0.52–1.93)	0.99
Surgery-Related Factors	Operative setting (Emergency vs. Elective)		2.29 (1.16–4.54)	0.017	2.15 (1.07–4.32)	0.032
	Stoma Location (Lower vs. Upper Abdomen)		6.15 (0.77–49.3)	0.086	5.80 (0.72–46.9)	0.099
	Stoma configuration (Loop vs. End)		1.42 (0.78–2.59)	0.250	1.38 (0.75–2.55)	0.300
	Surgical Approach (Open vs. Laparoscopic)		0.85 (0.52–1.38)	0.510	0.88 (0.53–1.46)	0.620
	Neoadjuvant ± Adjuvant therapy （vs. NO）		0.60 (0.32–1.12)	0.105	0.75 (0.37–1.52)	0.43
Model Diagnostics	Hosmer-Lemeshow test: *P* = 0.752 AUC: 0.782 (95% CI: 0.715–0.849)					

a : High: ≥24 kg/m^2^, Moderate: 18.5–23.9 kg/m^2^, Low: <18.5 kg/m^2.^

**Table 4:** Subgroup analysis of suture reinforcement on parastomal hernia incidence and early postoperative complications.

Subgroup Variable	Category	Total Patients (*N*)	Reinforcement Group Incidence(%) (n/N)	Conventional Group Incidence(%) (n/N)	Absolute Risk Reduction(%)	*P*-value
Smoking	With	104	9.5%(4/42)	27.4%(17/62)	17.9%	0.026
	Without	140	9.6%(5/52)	27.3%(24/88)	17.1%	0.013
Stoma Location	Upper Abdomen	15	14.3% (1/7)	12.5% (1/8)	−1.8%	0.733
	Lower Abdomen	229	9.2% (8/87)	28.2% (40/142)	19.0%	0.001
Operative setting	Emergency	45	19.0% (4/21)	70.8% (17/24)	51.8%	0.004
	Elective	199	6.8% (5/73)	19.0% (24/126)	12.2%	< 0.001
Stoma configuration & segment	Loop Colostomy	36	0.0% (0/10)	42.3% (11/26)	42.3%	0.013
	Loop Ileostomy	142	9.3% (5/54)	22.7% (20/88)	13.4%	0.041
	End Colostomy	42	14.3% (3/21)	38.1% (8/21)	23.8%	0.160
	End Ileostomy	24	11.1% (1/9)	13.3% (2/15)	2.2%	0.692
Suture	NO.7 Silk	142	10.9%(6/55)	26.4%(23/87)	15.5%	0.025
	1-0 Prolene	102	7.7%（3/39）	25.6%(18/63)	22.5%	0.012
Malignant Tumor	With	170	10.3%(7/68)	24.5%(25/102)	14.2%	0.020
	Without	74	7.7%(2/26)	33.3%(16/48)	25.6%	0.014
Neoadjuvant ± Adjuvant therapy	With	137	7.7%(4/52)	22.4%(19/85)	14.7%	0.046
	Without	107	11.9%(5/42)	33.8%(22/65)	21.9%	0.011
Early Postoperative Complications	Incisional Infection, *n* (%	14 (5.7)	6 (6.4)	8 (5.3)	−1.1%	0.732
	Stoma-Related Complications^a^, *n* (%)	7 (2.9)	3 (3.2)	4 (2.7)	−0.5%	1.000

a **:** Stoma-related complications：peristomal skin irritation/dermatitis, stoma ischemia, stoma retraction, stoma prolapse, stoma stenosis, peristomal infection/abscess, stoma bleeding, and parastomal fistula, among others, etc.

### 3.2 Risk-factor-analysis for parastomal hernia

Univariable analysis demonstrated that suture reinforcement (OR = 0.28, 95% CI 0.13–0.59, *P* = 0.001), elevated BMI (OR = 2.13, 95% CI 1.02–4.46, *P* = 0.044), and emergency surgery (OR = 2.29, 95% CI 1.16–4.54, *P* = 0.017) were significantly correlated with PSH occurrence.

Results of multivariable logistic regression and Firth-penalized regression were summarized. Multivariable logistic regression confirmed suture reinforcement as an independent protective factor for PSH (aOR = 0.29, 95% CI 0.13–0.65, *P* = 0.002). Emergency surgery (aOR = 2.15, 95% CI 1.07–4.32, *P* = 0.032) and increased BMI (aOR = 2.24, 95% CI 1.06–4.76, *P* = 0.035) represented independent risk factors. Firth-penalized logistic regression validated the stability of the above findings (suture reinforcement: OR = 0.31, 95% CI 0.14–0.68, *P* = 0.003) (Table 3).

### 3.3 Outcomes after propensity-score-matching

After 1:1-PSM, 94-pairs of patients were successfully matched; all-covariates achieved satisfactory-balance (SMD < 0.10). Within matched-cohort, PSH-incidence remained markedly lower in reinforcement-group compared with conventional-group (14.9% versus 31.9%, absolute-risk-reductio*n* = 17.0%, *P* = 0.006). Multivariable-regression based on matched-dataset still identified suture-reinforcement as independent-protective-factor (aOR = 0.35, 95%CI: 0.17-0.72, *P* = 0.004). The protective-effect was slightly attenuated compared with primary-unmatched-analysis (aOR 0.29 versus 0.35) (Table 2).

### 3.4 Interrupted time-series analysis

In the ITS analysis, segmented regression demonstrated that the suture reinforcement intervention was associated with both an immediate reduction in PSH incidence and a sustained attenuation of its upward trend over time. Specifically, the intervention was associated with a significant immediate level change (*β*2 = −0.074*β*2​=−0.074, 95% CI: −0.142 to −0.006, *P* = 0.034), indicating an approximately 7.4% absolute decrease in PSH incidence at the intervention time points. More importantly, the interaction term (*β*3 = −0.0031*β*3​=−0.0031, 95% CI: −0.0058 to −0.0004, *P* = 0.026) was significantly negative, suggesting that the suture reinforcement group experienced a slower monthly increase in PSH incidence over time compared with the conventional group. The model demonstrated good fit (R^2^ = 0.71). This ITS analysis helped to evaluate potential time-related confounding arising from alternating-month allocation and supported the robustness of our observational findings. Detailed outputs of segmented regression for ITS analysis are presented (Table 1, Figure 3).

**Figure 3:**
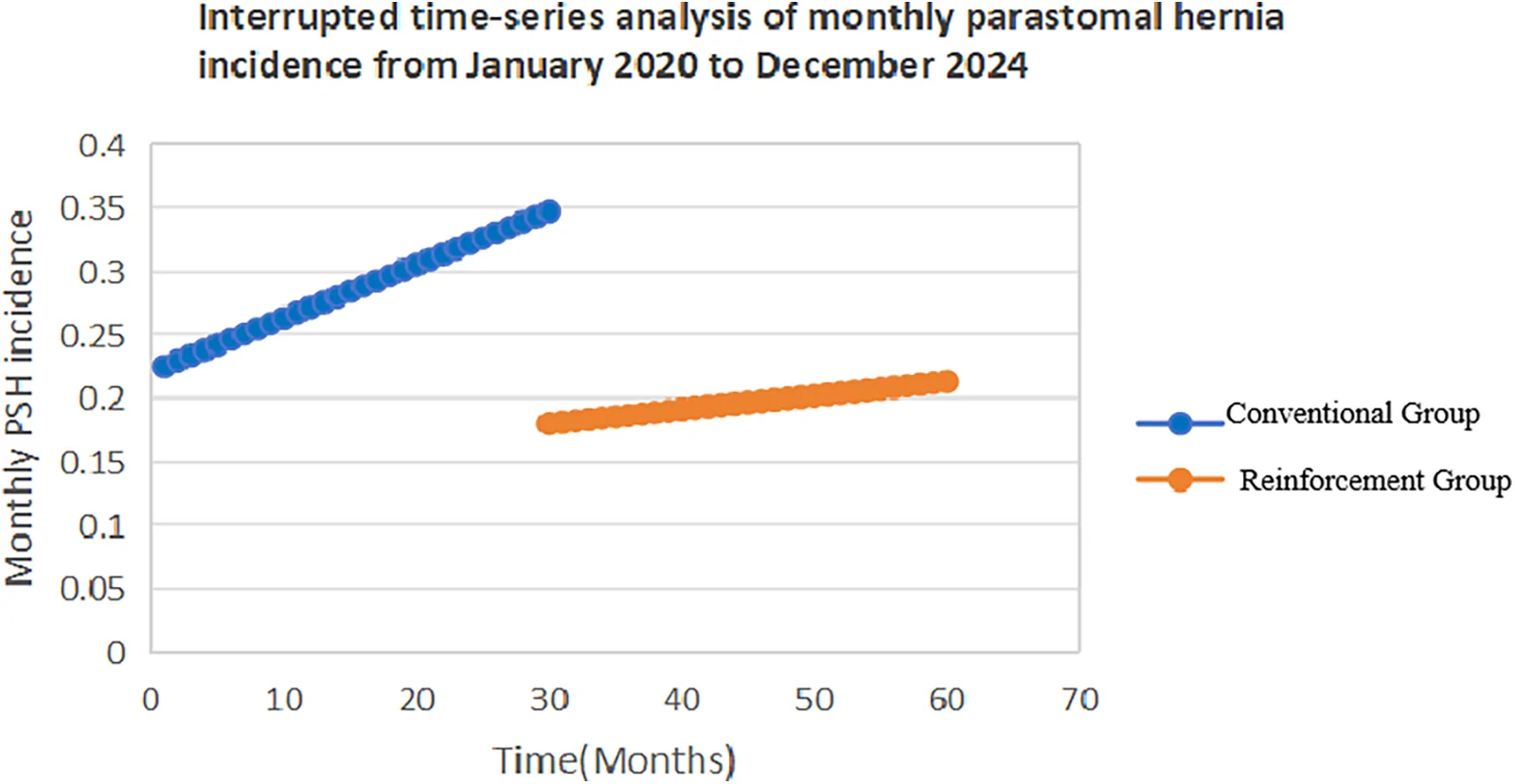
Interrupted time-series analysis monthly parastomal hernia incidence from January 2020 to December 2024.

### 3.5 Subgroup-analysis

Subgroup analyses were performed for subgroups with adequate sample sizes, and the results are presented in Table 4. In the lower abdominal stoma subgroup, the PSH rate was 9.2% (8/87) in the reinforcement group versus 28.2% (40/142) in the conventional group (*P* = 0.001), with an absolute risk reduction (ARR) of 19.0%. In the emergency surgery subgroup, PSH occurred in 19.0% (4/21) of the reinforcement group versus 70.8% (17/24) of the conventional group (*P* = 0.004), with an ARR of 51.8%. In the elective surgery subgroup, the PSH rate was 6.8% (5/73) versus 19.0% (24/126) (*P* < 0.001), with an ARR of 12.2%. For loop colostomy, no PSH events occurred in the reinforcement group (0/10) compared with 42.3% (11/26) in the conventional group (*P* = 0.013), with an ARR of 42.3%. For loop ileostomy, the PSH rate was 9.3% (5/54) versus 22.7% (20/88) (*P* = 0.041). For end colostomy, the rates were 14.3% (3/21) versus 38.1% (8/21) (*P* = 0.160); for end ileostomy, 11.1% (1/9) versus 13.3% (2/15) (*P* = 0.692). Subgroup analyses by smoking, suture material, malignant tumor, and adjuvant therapy also showed consistent protective effects (Table 4). Although the loop colostomy subgroup showed a striking intergroup difference, the small sample size and zero-event cell require cautious interpretation; these findings should be considered exploratory. Larger studies are needed to confirm efficacy in specific stoma types (Table 4).

### 3.6 Hernia-sac diameter and time-to-onset of PSH

Among the 50 patients who developed PSH during follow-up, the maximum hernia-sac diameter was significantly smaller in the reinforcement group (2.9 ± 1.2 cm) than in the conventional group (7.3 ± 2.7 cm, *P* < 0.001). The mean time to PSH detection was markedly longer in the reinforcement group (12.2 ± 7.1 months) than in the conventional group (6.7 ± 3.2 months, *P* = 0.001) (Table 5).

**Table 5:** Characteristics of patients who developed parastomal hernia.

Characteristics		Overall Hernia Patients (*n* = 50)	Reinforcement Group (*n* = 9)	Conventional Group(*n* = 41)	*P*-value
Parastomal Hernia-Related Features	Maximum hernia sac diameter (cm)^a^	6.4 ± 2.9	2.9 ± 1.2	7.3 ± 2.7	<0.001
	Time to hernia detection (months)	7.8 ± 4.2	12.2 ± 7.1	6.7 ± 3.2	0.001

a : CT measurement.

### 3.7 Survival-analysis

Kaplan–Meier analysis indicated that suture reinforcement significantly prolonged PSH-free survival time (log-rank *P* < 0.05). Obese patients and patients undergoing emergency surgery obtained obvious preventive benefit from this suture technique. The hazard-function curve is presented in the supplementary figures (Figures 4, 5).

**Figure 4:**
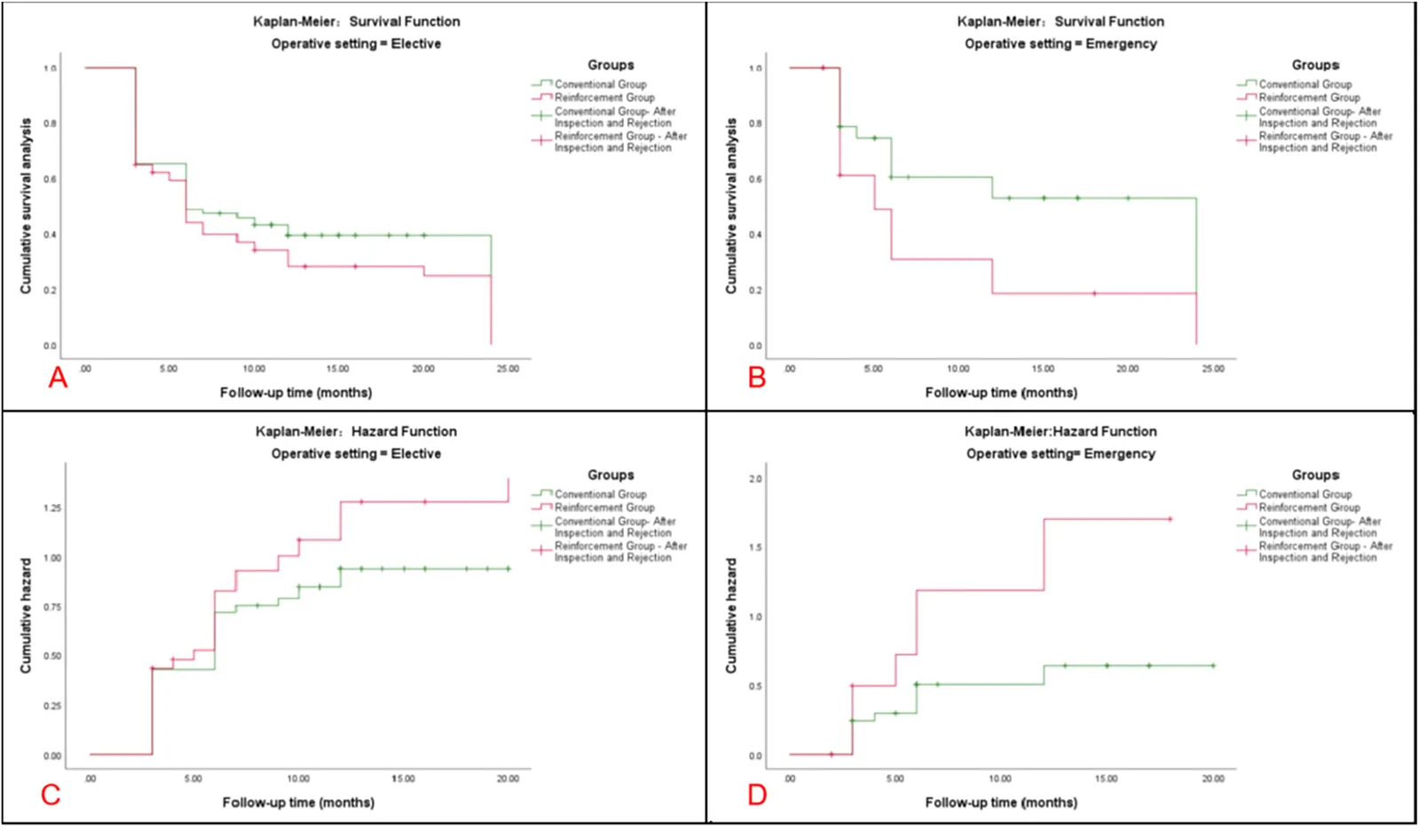
Comparison of survival curves and hazard curves across different surgical types (elective and emergency surgeries). **(A,B)** Survival curves for elective and emergency surgeries;**(C,D)** Hazard curves for elective and emergency surgeries. The results demonstrate that suture reinforcement significantly prolongs parastomal hernia-free survival and reduces the incidence of parastomal hernia in both elective and emergency surgery patients. Moreover, this protective effect is particularly pronounced in patients undergoing high-risk emergency procedures.

**Figure 5:**
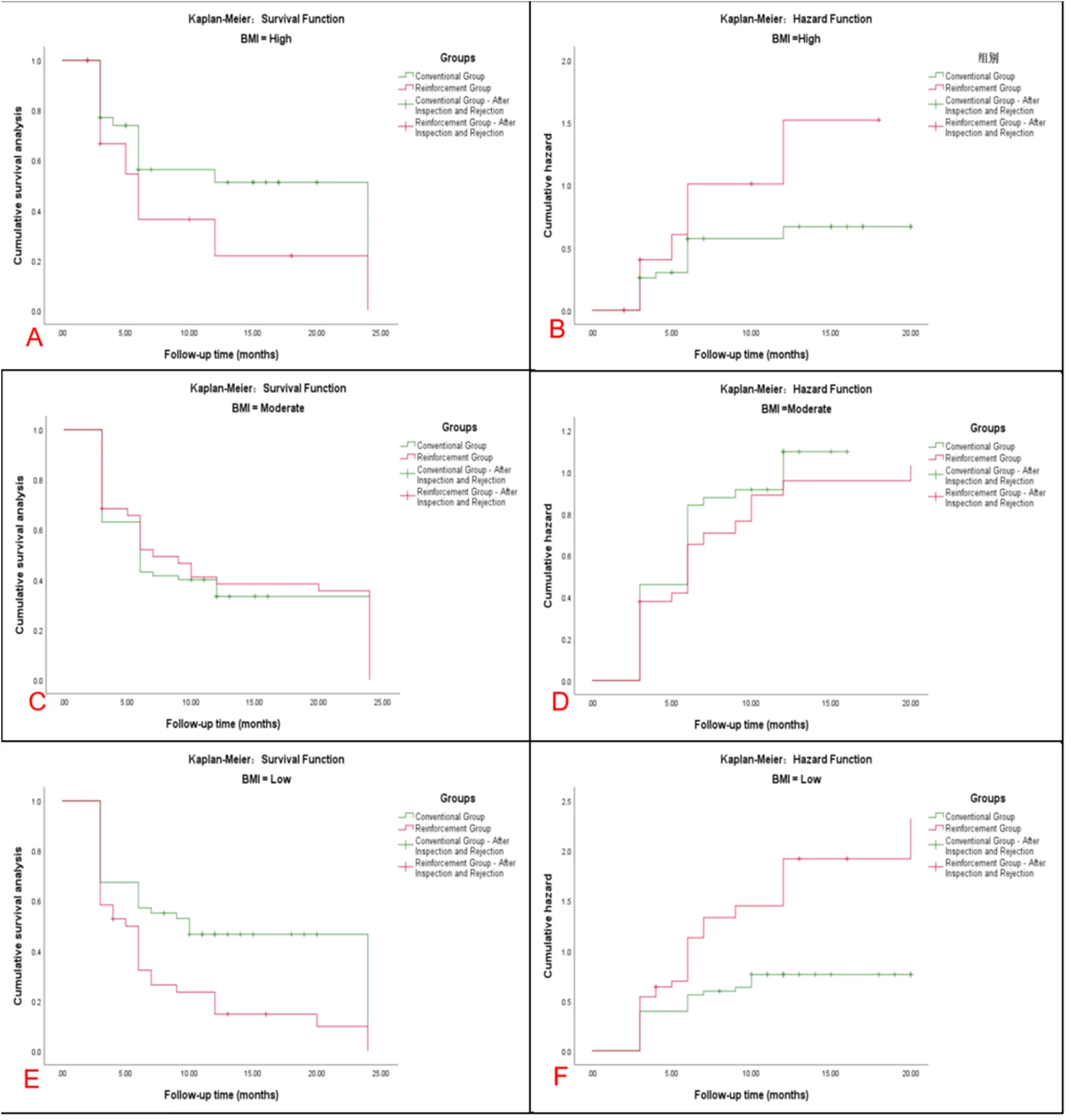
Comparison of survival curves and hazard curves across different BMI categories. **(A,C,E)** Survival curves for high, moderate, and low BMI subgroups; **(B,D,F)** Hazard curves for high, moderate, and low BMI subgroups. The results demonstrate that suture reinforcement significantly prolongs parastomal hernia-free survival and reduces the incidence of parastomal hernia in patients with high BMI. Moreover, this protective effect is particularly pronounced in high-risk patients with elevated BMI.

### 3.8 Regression-model-performance

The Hosmer–Lemeshow test yielded *P* = 0.752, suggesting satisfactory model goodness-of-fit. The AUC reached 0.782 (95% CI: 0.715–0.849), indicating moderate discriminatory power of the multivariable logistic regression model (Table 3).

## 4 Discussion

Despite continuous advances in abdominal wall surgery, PSH remains a persistent clinical challenge in the fields of colorectal and abdominal wall surgery worldwide (14). The pathogenesis of PSH primarily involves the disruption of the mechanical integrity of the abdominal wall, a process that can be precisely described by Laplace's law: the wall tension (T) acting on an abdominal wall defect is directly proportional to the product of intra-abdominal pressure (P) and defect radius (r), i.e., T ∝ *P* × r. In conventional enterostomy, the bowel traverses the rectus abdominis muscle and its sheath, thereby compromising the continuity of the abdominal wall and creating natural areas of weakness at the superior and inferior poles of the stoma. Given the longitudinal orientation of the rectus abdominis fibres, the defect bears the greatest Laplace tension along the vertical axis (i.e., the superior-inferior direction), which constitutes the predisposed site of weakness; horizontal suturing can counteract this vertical tension. Under conditions of chronically elevated intra-abdominal pressure (e.g., obesity, chronic cough, and constipation), the defect gradually enlarges along the direction of the muscle fibres, ultimately leading to the development and progression of PSH (15).

According to clinical guidelines, the primary principle in PSH repair is to narrow the defect and perform full-thickness suture reinforcement of the rectus abdominis (16). Based on this principle, the rectus abdominis complex reinforcement technique used in this study—passing non-absorbable sutures through the entire thickness of the rectus abdominis complex at the superior and inferior poles of the stoma—mechanically stabilizes the stoma aperture and limits its progressive enlargement due to tissue relaxation and repeated fluctuations in intra-abdominal pressure. This manoeuvre reduces the effective radius of the potential abdominal wall defect and, according to Laplace's law, directly lowers local tension. This technique replaces passive tissue adhesion with mechanical reconstruction of the rectus abdominis and its sheath complex, strengthens the pressure-resisting capacity of the peristomal abdominal wall, and thereby reduces the incidence of PSH.

Consistent with this mechanism, the results of this retrospective cohort study show that prophylactic full-thickness reinforcement suturing of the rectus abdominis complex during intestinal ostomy creation significantly reduces the incidence of PSH, delays its occurrence, and decreases the hernia sac diameter. Multivariable logistic regression analysis demonstrated that full-thickness reinforcement suturing is an independent protective factor against PSH (aOR = 0.29, 95% CI: 0.13–0.65, *P* = 0.002). After adjusting for age, sex, surgical approach, stoma location, and comorbidities, the protective effect remained stable, suggesting that the observed benefit derives from the mechanical reinforcement itself rather than from baseline differences between groups. Meanwhile, high BMI (aOR = 2.24, *P* = 0.035) and emergency surgery (aOR = 2.15, *P* = 0.032) were identified as independent predictors of PSH, a finding consistent with recent systematic reviews and population-based studies (17). These two patient groups generally have higher baseline intra-abdominal pressure, poorer abdominal wall condition, and greater difficulty in stoma construction, making them key targets for clinical prevention.

Of note, reinforcement suturing maintained a consistent protective effect in these high-risk subgroups. Patients with high BMI and those undergoing emergency surgery are at exceptionally high risk of PSH. Although their pathophysiologic mechanisms differ, both promote PSH by increasing effective stomal aperture size and imposing greater intra-abdominal pressure load (17). Patients with high BMI have excess subcutaneous and visceral fat, attenuated abdominal musculature, and limited surgical exposure, often requiring a larger aperture for safe bowel exteriorization; persistently elevated intra-abdominal pressure further drives defect enlargement (18). Emergency stomas are frequently fashioned for acute indications such as obstruction or perforation, with bowel congestion and oedema necessitating a larger aperture to avoid ischaemia; as oedema resolves postoperatively, the stomal defect becomes relatively enlarged, creating an anatomical predisposition to herniation (19). In our subgroup analysis, the conventional group had a PSH rate of 70.8% in emergency patients, compared with only 19.0% in the reinforcement group, representing an absolute risk reduction of 51.8%. Reinforcement suturing also reduced PSH rates among patients with high BMI. These findings suggest that full-thickness suturing compensates for the anatomical and physiological vulnerabilities of these high-risk populations by stabilizing the stomal aperture and counteracting dynamic intra-abdominal pressure changes.

In the loop colostomy subgroup (*n* = 36), no PSH events occurred in the reinforcement group (0/10), compared with 42.3% (11/26) in the conventional group. While this suggests a potential benefit of prophylactic full-thickness suturing for loop colostomy, the small sample size—especially the zero-event cell in the reinforcement group—requires cautious interpretation. Complete separation may have influenced logistic regression estimates, and although Firth's penalized method was used in the primary analysis, the subgroup was not similarly adjusted. These findings should therefore be considered exploratory, and larger studies are needed to confirm efficacy in loop colostomy.

Prophylactic mesh reinforcement is currently the most widely investigated strategy for PSH prevention (20), with multiple meta-analyses confirming reduced hernia rates. However, current international guidelines do not support its routine use (21). As a permanent foreign body, mesh carries risks of surgical site infection, intestinal erosion, chronic pain, and stoma dysfunction, occasionally requiring reintervention (22). In contrast, full-thickness reinforcement suturing involves no implanted material and thus avoids foreign body-related complications. In our study, the reinforcement group showed no increase in stoma ischaemia, retraction, or infection, supporting favourable safety. Furthermore, meshes—particularly biologic or specialized PSH meshes—are costly and poorly accessible in many settings (23); reinforcement suturing uses only standard non-absorbable suture material, requires no additional consumables, and imposes minimal economic burden. Mesh placement demands meticulous dissection and precise fixation around the stoma and carries a steep learning curve; reinforcement suturing integrates seamlessly into standard stoma creation, has a low technical barrier, and can be readily adopted.

Beyond mesh reinforcement, lateral pararectal (pararectus abdominis) stoma placement represents another preventive strategy. The PATRASTOM tria (24)l, the only randomized controlled trial comparing lateral pararectal with transrectus abdominis stoma placement, found no significant difference in PSH incidence (5/27 vs. 4/29, *P* = 0.725); however, this trial was limited by a small sample size and short follow-up. A Cochrane review (25) similarly found uncertain evidence (RR = 1.34, 95% CI 0.40–4.48; low-quality evidence). Importantly, our technique does not alter the anatomical stoma location but mechanically reinforces the transrectus abdominis route, targeting the fascial defect itself. Therefore, lateral pararectal placement and suture reinforcement may be complementary rather than competing strategies, and future studies should explore whether combining them provides additive benefit.

Although smoking and adjuvant therapy are recognized factors affecting postoperative outcomes, neither was independently associated with PSH in this cohort. This may be due to the limited sample size, binary smoking classification, lack of detailed oncological treatment data, and the predominantly mechanical pathogenesis of PSH. Larger prospective studies incorporating smoking dose/cessation and treatment details are needed.

### 4.1 Limitations

This study is a single-centre retrospective cohort study with alternating-month allocation rather than randomisation. Although group assignment was predetermined by calendar month and independent of individual patient risk profiles, temporal confounding factors could not be completely eliminated. While we employed interrupted time-series (ITS) analysis to explicitly model time-related confounding, the study remains retrospective and non-randomised. Although ITS is robust against secular trends, it cannot fully exclude unmeasured time-varying confounders that coincide with the alternating-month schedule. Furthermore, the ITS model was aggregated at the monthly level, and individual-level time-dependent covariates were not captured. Despite the use of ITS to assess secular-trend confounding, seasonal variation in case mix could not be completely ruled out. Therefore, our findings should be interpreted as strong observational evidence rather than definitive causal proof, and prospective randomised trials are warranted to validate them.

Second, a competing-risks statistical model was not used to handle stoma-reversal events. A considerable proportion of loop ileostomy patients underwent early stoma closure within 3–6 months postoperatively, shortening the actual at-risk follow-up duration for PSH occurrence. Consequently, the Kaplan–Meier method may overestimate the cumulative PSH incidence. Our results are more applicable to patients with a permanent stoma or those without a planned early reversal.

Third, several subgroups had relatively small sample sizes. Moreover, mild asymptomatic PSH might escape detection even with contrast-enhanced CT. Long-term follow-up data for patients with unreversed temporary stomas are still being accumulated. We plan to launch a prospective multicentre randomised controlled trial with a standardised imaging evaluation protocol to further validate the long-term efficacy and generalisability of this suture-reinforcement technique.

## 5 Conclusion

In this retrospective-cohort-study, this implant-free suture-reinforcement-technique was associated with reduced-PSH-incidence. Secondary-observations indicated delayed-hernia-onset and decreased-hernia-severity. This procedure is technically-simple and requires no additional-implant-cost, and may confer preventive-benefit for high-risk-patients such as obese-individuals and patients undergoing emergency-enterostomy. Prospective-randomized-studies are required to confirm its long-term-efficacy and external-generalizability.

## Data Availability

The datasets presented in this article are not readily available because the dataset contains patient clinical and imaging data and cannot be made publicly available due to patient privacy protection and institutional ethical restrictions. Access is limited to the research team and the study sponsor; anonymized aggregate data may be available upon reasonable request to the corresponding author, subject to institutional approval. Requests to access the datasets should be directed to Ethics Committee of Shengzhou People's Hospital (Shengzhou Branch of the First Affiliated Hospital of Zhejiang University School of Medicine, the Shengzhou Hospital of Shaoxing University).
